# Short and Long-Term Outcome of 75 or Over Aged Patients Admitted to Intensive Care Unit (ICU): A Single Center, Observational Study

**DOI:** 10.1186/2197-425X-3-S1-A528

**Published:** 2015-10-01

**Authors:** D Lathyris, M Bogiatzopoulos, A Boutou, F Renta, E Antipa, E Chassou, E Antoniadou

**Affiliations:** Gennimatas General Hospital,Thessaloniki, Greece, Intensive Care Unit, Thessaloniki, Greece

## Introduction

Age is considered an independent risk factor for short and long-term mortality of elderly ICU patients, when very elderly (≥85 years) and mid-elderly (75-84 years) populations are compared to young elderly (65-74 years) ones. However, it is not yet clear if this trend remains when very elderly are compared to mid-elderly populations.

## Objectives

a) to compare short (ICU and hospital) and long-term (6-months and 1-year) mortality of mid-elderly to very elderly patients and

b) to evaluate the influence of patients' characteristics on mortality.

## Methods

Single center, retrospective, observational study in an 8-bed adult general ICU (January 2011-June 2014). Patients ≥75 years were divided into two age-groups, 75-84 and ≥85 years old. Characteristics on ICU admission were recorded. Patients hospitalized ≤48 hours were excluded. ICU, hospital, 6-months and 1-year after hospital discharge mortality were calculated. Univariate analysis for categorical variables was performed using Pearson’s x2 or Fisher test and Student t-test for continuous data. Multivariate analysis of the time to ICU mortality was calculated using Cox regression model and for hospital, 6-months and 1-year mortality using logistic regression analysis. P value < 0.05 was considered significant.

## Results

244 patients were included, 195 in the 75-84 and 49 in the ≥85 years group. Mortality rates for the two groups were: ICU, 74/195 (37.9%) vs. 24/49 (49%) (p = 0.08), hospital, 23/121 (19%) vs. 5/25 (20%) (p = 0.90), 6-months, 15/95 (17%) vs. 4/20 (20%) (p = 0.74) and 1-year, 22/80 (27.5%) vs. 7/16 (20%) (p = 0.19), respectively. in multivariable analysis, patients with malignancy as reason for ICU admission had increased ICU mortality risk (HR:1.45; CI 95%, 1.02-2.05; p = 0.03). Patients with higher APACHE II score on ICU admission had more important 1-year mortality risk (OR:1.09; CI 95%, 1.016-1.16; p = 0.015).

## Conclusions

More than one third of our ICU patients were mid- or very elderly. No difference was observed between the two age-groups considering short or long-term mortality. Malignancy as a reason for ICU admission and APACHE II score negatively influenced the ICU and 1-year mortality, respectively.

## References

[1] Fuchs L, Novack V, McLennan S, Celi LA, Baufield Y, Park S (2014). Trends in severity of illness on ICU admission and mortality among the elderly. PLoS One.

[2] Fuchs L, Chronaki CE, Park S, Novack V, Baumfield Y, Scott D (2012). ICU admission characteristics and mortality rates among elderly and very elderly patients. Intensive Care Med.

